# Supplementary material for: Machine Learning Accelerates Crystallization for Structure Determination
Source: Angew Chem Int Ed Engl. 2026 May 4;65(25):e1218503. doi: 10.1002/anie.1218503 (PMC13266958; doi:10.1002/anie.1218503)

checkCIF/PLATON report

Structure factors have been supplied for datablock(s) lcz031

THIS REPORT IS FOR GUIDANCE ONLY. IF USED AS PART OF A REVIEW PROCEDURE FOR PUBLICATION, IT SHOULD NOT REPLACE THE EXPERTISE OF AN EXPERIENCED CRYSTALLOGRAPHIC REFEREE.

No syntax errors found. CIF dictionary Interpreting this report

Datablock: lcz031

Bond precision:	C-C = 0.0104 A		Wavelength=1.54184
Cell:	a=13.6963(2)	b=17.6966(2)	c=19.9309(4)
	alpha=91.036(1)	beta=93.360(1)	gamma=106.910(1)
Temperature:	100 K		

	Calculated	Reported
Volume	4610.87(13)	4610.87(13)
Space group	P -1	P -1
Hall group	-P 1	-P 1
Moiety formula	C37 H27 Ag3 F18 N6 O6 [+ solvent]	C15 H3 Ag3 F18 N6, 2(C11 H12 O3)
Sum formula	C37 H27 Ag3 F18 N6 O6 [+ solvent]	C37 H27 Ag3 F18 N6 O6
Mr	1317.26	1317.25
Dx,g cm-3	1.898	1.898
Z	4	4
Mu (mm-1)	11.258	11.258
F000	2568.0	2568.0
F000'	2579.73	
h,k,lmax	17,22,25	17,22,25
Nref	19886	18889
Tmin,Tmax	0.231,0.259	0.556,1.000
Tmin'	0.148	

Correction method= # Reported T Limits: Tmin=0.556 Tmax=1.000
AbsCorr = MULTI-SCAN

Data completeness= 0.950 Theta(max)= 78.694

R(reflections)= 0.0589(15905) wR2(reflections)= 0.1464(18889)

S = 1.076 Npar= 1269

The following ALERTS were generated. Each ALERT has the format
test-name_ALERT_alert-type_alert-level.
Click on the hyperlinks for more details of the test.

Alert level C

```
PLAT213_ALERT_2_C Atom F016            has ADP max/min Ratio .....        3.2 prolat
PLAT220_ALERT_2_C NonSolvent   Resd 1  C   Ueq(max)/Ueq(min) Range        3.7 Ratio
PLAT220_ALERT_2_C NonSolvent   Resd 1  F   Ueq(max)/Ueq(min) Range        5.1 Ratio
PLAT230_ALERT_2_C Hirshfeld Test Diff for    O008      --C01N     .       5.2 s.u.
PLAT234_ALERT_4_C Large Hirshfeld Difference F02R      --C03V     .      0.16 Ang.
PLAT234_ALERT_4_C Large Hirshfeld Difference C034      --C03M     .      0.17 Ang.
PLAT342_ALERT_3_C Low Bond Precision on  C-C Bonds ...............    0.01044 Ang.
PLAT906_ALERT_3_C Large K Value in the Analysis of Variance ......      4.139 Check
PLAT911_ALERT_3_C Missing FCF Refl Between Thmin & STh/L=    0.600         94 Report
              -12 18  0, -11 19  0,   8-19  1,  12-18  1,  12-17  1,  13-17  1,
               -1 -1  1,   1 -1  1,   1  0  1,  -1  1  1,   0  1  1,   1  1  1,
                4 18  1,  12-18  2,  11-17  2,  12-17  2,  13-17  2,   9 -8  2,
                0  0  2,   0  1  2,   0  2  2,  -8 20  2,  -6 21  2,  11-18  3,
               12-17  3,  13-16  3,   0  1  3,   0  2  3,  12-17  4,  14-15  4,
                   (   64 More Missing: see the .ckf listing file)
PLAT971_ALERT_2_C Check Calcd Resid. Dens.  1.44Ang From C03V          2.49 eA-3
```

Alert level G

```
PLAT002_ALERT_2_G Number of Distance or Angle Restraints on AtSite          3 Note
PLAT003_ALERT_2_G Number of Uiso or U(i,j)  Restrained non-H-Atoms          7 Report
PLAT042_ALERT_1_G Calc. and Reported MoietyFormula Strings  Differ     Please Check
              Calc: C37 H27 Ag3 F18 N6 O6
              Rep.: C15 H3 Ag3 F18 N6, 2(C11 H12 O3)
PLAT083_ALERT_2_G SHELXL Second Parameter in WGHT  Unusually Large      36.47 Why ?
PLAT154_ALERT_1_G The s.u.'s on the Cell Angles are Equal ..(Note)      0.001 Degree
PLAT172_ALERT_4_G The CIF-Embedded .res File Contains DFIX Records          3 Report
PLAT186_ALERT_4_G The CIF-Embedded .res File Contains ISOR Records          2 Report
PLAT242_ALERT_2_G Low    'MainMol' Ueq as Compared to Neighbors of       C02P Check
PLAT242_ALERT_2_G Low    'MainMol' Ueq as Compared to Neighbors of       C02Q Check
PLAT242_ALERT_2_G Low    'MainMol' Ueq as Compared to Neighbors of       C035 Check
PLAT242_ALERT_2_G Low    'MainMol' Ueq as Compared to Neighbors of       C03B Check
PLAT242_ALERT_2_G Low    'MainMol' Ueq as Compared to Neighbors of       C03E Check
PLAT242_ALERT_2_G Low    'MainMol' Ueq as Compared to Neighbors of       C03V Check
PLAT242_ALERT_2_G Low    'MainMol' Ueq as Compared to Neighbors of       C02G Check
PLAT242_ALERT_2_G Low    'MainMol' Ueq as Compared to Neighbors of       C036 Check
PLAT242_ALERT_2_G Low    'MainMol' Ueq as Compared to Neighbors of       C03A Check
PLAT242_ALERT_2_G Low    'MainMol' Ueq as Compared to Neighbors of       C03D Check
```

```
PLAT242_ALERT_2_G Low    'MainMol' Ueq as Compared to Neighbors of       C03J Check
PLAT242_ALERT_2_G Low    'MainMol' Ueq as Compared to Neighbors of       C03S Check
PLAT343_ALERT_2_G Unusual sp3      Angle Range in Main Residue for       C03V Check
PLAT434_ALERT_2_G Short Inter HL..HL Contact F00C     ..F01J     .        2.82 Ang.
                                                 1-x,1-y,-z  =      2_665 Check
PLAT434_ALERT_2_G Short Inter HL..HL Contact F015     ..F01M     .        2.63 Ang.
                                                      x,y,z  =      1_555 Check
PLAT605_ALERT_4_G Largest Solvent Accessible VOID in the Structure         281 A**3
PLAT720_ALERT_4_G Number of Unusual/Non-Standard Labels .........          190 Note
              Ag02    Ag03    Ag04    Ag05    Ag06    O007    O008    F00A
              F00B    F00C    F00D    O00E    O00F    F00G    F00H    O00I
              F00J    F00K    O00L    F00M    N00N    N00O    F00P    N00Q
              F00R    F00S    N00T    F00U    N00V    F00W    F00X    O00Y
              F00Z    N010    N011    N012    N013    F014    F015    F016
              F017    N018    N019    F01A    F01B    O01C    N01D    O01E
              F01F    F01G    O01H    F01I    F01J    F01K    F01L    F01M
              C01N    C01O    C01P    C01Q    H01Q    C01R    F01S    C01T
              C01U    H01U    O01V    C01W    C01X    O01Y    C01Z    C020
              H02A    H02B    C021    C022    H022    C023    H023    C024
              C025    F026    C027    C028    C029    H029    C02A    H02F
              C02B    H02C    C02C    H02D    H02E    C02D    C02E    C02F
              C02G    C02H    C02I    C02J    C02K    C02L    H02L    C02M
              H02M    C02N    C02O    C02P    C02Q    F02R    C02S    C02T
              H02T    C02U    C02V    H02I    H02J    C02W    H02G    H02H
              C02X    C02Y    C02Z    H02K    H02N    C030    H030    C031
              C032    H03Q    H03R    H03S    C033    C034    H034    C035
              C036    C037    H03G    H03H    H03I    C038    H03J    H03K
              C039    H03A    H03B    H03C    C03A    C03B    F03C    C03D
              C03E    C03F    H03Y    C03G    H03Z    C03H    C03I    H03T
              H03U    C03J    C03K    H03L    H03M    H03N    C03L    C03M
              C03N    H03     Ha      C03P    H03O    H03P    C03Q    H03V
              H03W    H03X    F03R    C03S    C03U    H03D    H03E    H03F
              C03V    C03W    Hb      Hc      F03Y    F040
PLAT860_ALERT_3_G Number of Least-Squares Restraints ............           45 Note
PLAT868_ALERT_4_G ALERTS Due to the Use of _smtbx_masks Suppressed           ! Info
PLAT883_ALERT_1_G Absent Datum for _atom_sites_solution_primary ..     Please Do !
PLAT910_ALERT_3_G Missing FCF Reflection(s) Below Theta(Min)[Deg]=       3.37 Note
                0  1  0,   0  0  1,
PLAT912_ALERT_4_G Missing # of FCF Reflections Above STh/L=  0.600         899 Note
PLAT933_ALERT_2_G Number of HKL-OMIT Records in Embedded .res File          52 Note
              -15 16  1, -14 17  0, -14 17  1, -13 18  1, -13 18  5, -12 18  0,
               -9 18  7,  -8 18  5,  -8 20  2,  -6 20  4,   0  1  1,   0  1  2,
                0  1  3,   0  2  2,   0  2  3,   1  0  1,   2-20  7,   2-18 10,
                3-18  9,   3-17  9,   3-17 10,   4-21  5,   4-21  7,   4-18 10,
                6-22  5,   6-18 13,   6-16 14,   8-21  3,   8-21  4,   8-21  5,
                8-21  6,   8-19  1,   8-19  9,   8-17  7,   9-21  3,   9-21  4,
                9-20  5,   9-20  6,   9-19  7,   9-18  9,   9-15  9,   9-14  7,
                9-11  9,  11-18  3,  11-17  2,  12-18  1,  12-17  1,  12-17  2,
               12-17  3,  13-17  1,
PLAT941_ALERT_3_G Average HKL Measurement Multiplicity ..........          2.5 Low
PLAT969_ALERT_5_G The 'Henn et al.' R-Factor-gap value ..........        2.851 Note
              Predicted wR2: Based on SigI**2  5.14 or SHELX Weight 13.61
PLAT978_ALERT_2_G Number C-C Bonds with Positive Residual Density.           0 Info
```

---

0 **ALERT level A** = Most likely a serious problem - resolve or explain

0 **ALERT level B** = A potentially serious problem, consider carefully

```
 10 ALERT level C = Check. Ensure it is not caused by an omission or oversight
 33 ALERT level G = General information/check it is not something unexpected

  3 ALERT type 1 CIF construction/syntax error, inconsistent or missing data
 25 ALERT type 2 Indicator that the structure model may be wrong or deficient
  6 ALERT type 3 Indicator that the structure quality may be low
  8 ALERT type 4 Improvement, methodology, query or suggestion
  1 ALERT type 5 Informative message, check
```

Validation response form

Please find below a validation response form (VRF) that can be filled in and pasted into your CIF.

```
# start Validation Reply Form
_vrf_PLAT213_lcz031
;
PROBLEM: Atom F016            has ADP max/min Ratio .....        3.2 prolat
RESPONSE: ...
;
_vrf_PLAT220_lcz031
;
PROBLEM: NonSolvent   Resd 1  C   Ueq(max)/Ueq(min) Range        3.7 Ratio
RESPONSE: ...
;
_vrf_PLAT230_lcz031
;
PROBLEM: Hirshfeld Test Diff for    O008     --C01N     .        5.2 s.u.
RESPONSE: ...
;
_vrf_PLAT234_lcz031
;
PROBLEM: Large Hirshfeld Difference F02R     --C03V     .       0.16 Ang.
RESPONSE: ...
;
_vrf_PLAT342_lcz031
;
PROBLEM: Low Bond Precision on  C-C Bonds ..............    0.01044 Ang.
RESPONSE: ...
;
_vrf_PLAT906_lcz031
;
PROBLEM: Large K Value in the Analysis of Variance ......      4.139 Check
RESPONSE: ...
;
_vrf_PLAT911_lcz031
;
PROBLEM: Missing FCF Refl Between Thmin & STh/L=    0.600         94 Report
RESPONSE: ...
;
_vrf_PLAT971_lcz031
;
PROBLEM: Check Calcd Resid. Dens.  1.44Ang From C03V           2.49 eA-3
RESPONSE: ...
;
# end Validation Reply Form
```

It is advisable to attempt to resolve as many as possible of the alerts in all categories. Often the minor alerts point to easily fixed oversights, errors and omissions in your CIF or refinement strategy, so attention to these fine details can be worthwhile. It is up to the individual to critically assess their own results and, if necessary, seek expert advice.

PLATON version of 17/09/2025; check.def file version of 17/09/2025

duplicate check

No duplication found

Datablock lcz031 - ellipsoid plot

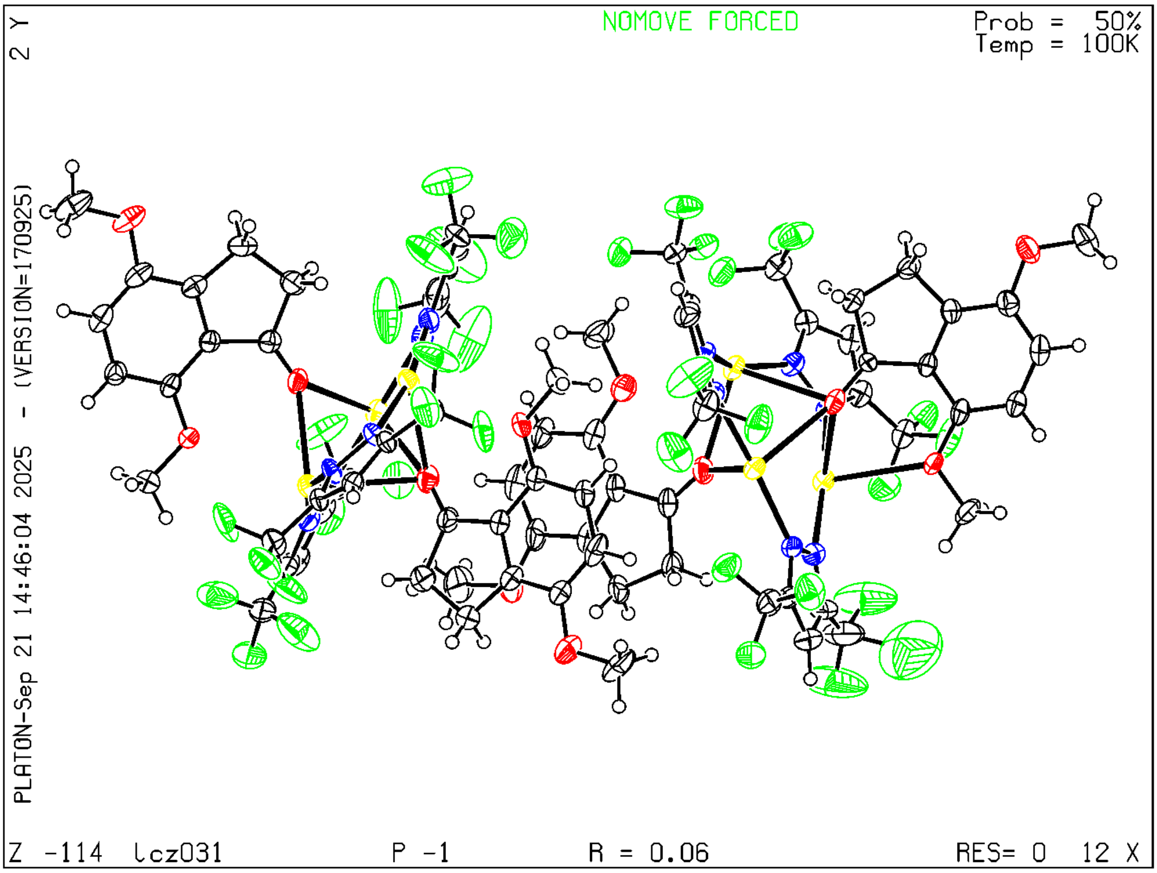

Supplement: Supplementary file 2 — Supporting File 2: anie72462‐sup‐0002‐DataFile.zip. [file ANIE-65-e1218503-s002.zip › supplementary_materials_crystallographic_data submit.zip!/cif_and_checkcif(1-114)/Ag3Pz3_31/Ag3Pz3_31_checkcif.pdf]
